# Nanodrug Transmembrane Transport Research Based on Fluorescence Correlation Spectroscopy

**DOI:** 10.3390/membranes11110891

**Published:** 2021-11-19

**Authors:** Xinwei Gao, Yanfeng Liu, Jia Zhang, Luwei Wang, Yong Guo, Yinru Zhu, Zhigang Yang, Wei Yan, Junle Qu

**Affiliations:** Center for Biomedical Photonics, Key Laboratory of Optoelectronic Devices and Systems of Ministry of Education and Guangdong Province, College of Physics and Optoelectronic Engineering, Shenzhen University, Shenzhen 518060, China; 1910454012@email.szu.edu.cn (X.G.); szu_liuyanfeng@163.com (Y.L.); julyzhang2021@163.com (J.Z.); wanglowell@szu.edu.cn (L.W.); 1800284004@email.szu.edu.cn (Y.G.); 2150453014@email.szu.edu.cn (Y.Z.); zhgyang@szu.edu.cn (Z.Y.); jlqu@szu.edu.cn (J.Q.)

**Keywords:** carbon dots, fluorescence correlation spectroscopy, doxorubicin, transmembrane transport

## Abstract

Although conventional fluorescence intensity imaging can be used to qualitatively study the drug toxicity of nanodrug carrier systems at the single-cell level, it has limitations for studying nanodrug transport across membranes. Fluorescence correlation spectroscopy (FCS) can provide quantitative information on nanodrug concentration and diffusion in a small area of the cell membrane; thus, it is an ideal tool for studying drug transport across the membrane. In this paper, the FCS method was used to measure the diffusion coefficients and concentrations of carbon dots (CDs), doxorubicin (DOX) and CDs-DOX composites in living cells (COS7 and U_2_OS) for the first time. The drug concentration and diffusion coefficient in living cells determined by FCS measurements indicated that the CDs-DOX composite distinctively improved the transmembrane efficiency and rate of drug molecules, in accordance with the conclusions drawn from the fluorescence imaging results. Furthermore, the effects of pH values and ATP concentrations on drug transport across the membrane were also studied. Compared with free DOX under acidic conditions, the CDs-DOX complex has higher cellular uptake and better transmembrane efficacy in U_2_OS cells. Additionally, high concentrations of ATP will cause negative changes in cell membrane permeability, which will hinder the transmembrane transport of CDs and DOX and delay the rapid diffusion of CDs-DOX. The results of this study show that the FCS method can be utilized as a powerful tool for studying the expansion and transport of nanodrugs in living cells, and might provide a new drug exploitation strategy for cancer treatment in vivo.

## 1. Introduction

Cancer is a very serious disease threatening human health, with ever-increasing morbidity and mortality year by year. At present, chemotherapy with molecular drugs such as DOX is considered one of the most important methods for the treatment of malignant tumors. However, unmodified molecular drugs generally have poor tumor-targeting ability and short retention time, which may cause severe side effects and reduce their efficacy [1,2,3]. To address these issues, drug nanocarrier systems with the ability of improving the therapeutic efficiency and reducing side effects have been designed and applied for tumor theranostic studies [4,5,6]. Recently, CDs emerged as a class of promising nanocarriers owing to their small size, high bio-compatibility, hydrophilicity and responsive surface functionalities [7,8,9,10]. By cooperating with CDs to form nanocomposites, molecular drugs may gain higher transmembrane trafficking efficiency specifically in the tumor regions and hence, achieving improved treatment efficacy and causing less adverse effects.

To date, researchers have made some progress in the research of CD-loaded DOX drug delivery systems. For example, the CDs-DOX complex prepared by Yuan et al. exhibited pH-dependent DOX release behavior, and showed low toxicity in cell and in vivo experiments [11]. Dan et al. studied a pH-responsive fluorescent therapeutic drug delivery system, and found that carbon dots (CDs) loaded with DOX can be used for intracellular drug delivery and tracking of human gastric cancer cells, but quantitative results from this system are lacking [12]. Wang et al. developed a CDs-DOX drug carrier system and imaging probes targeting the nucleus, which have excellent targeting and imaging properties [13]. Zhang et al. conceived a luminescent and pH-sensitive nanocarrier by combining CDs with DOX lipid-coated calcium phosphate (LCP) nanoparticles for the delivery of antitumor drugs, which improved the efficacy of the drugs in the tumor microenvironment with lower pH values [14]. Despite the rapid development in the material design, in-depth studies on the transmembrane delivery process of these CDs-DOX drug carrier systems remained a daunting challenge. This is partially due to the lack of competent biophotonic tools in relative studies: so far, the delivery of CDs-DOX systems has only been studied by fluorescence intensity imaging methods [15,16], which failed to provide quantitative results on the real-time specific drug concentrations in living cells, let alone the kinetic character of transmembrane drug delivery under different conditions.

FCS was first introduced by Magde et al. in 1972 [17]. FCS is an extremely sensitive analysis tool for the measurement of fluorescence fluctuations of a very small number of molecules in a small volume (~1 femtoliter). These fluctuations act as carriers of information and are decoded during the autocorrelation measurement to obtain various physicochemical information of interest, such as the diffusion coefficient and concentration. As a result, FCS can be applied for the non-invasive imaging study of mass transportation across cellular membranes with high sensitivity, high precision, high spatiotemporal resolutions [18,19].

**Figure 1:**
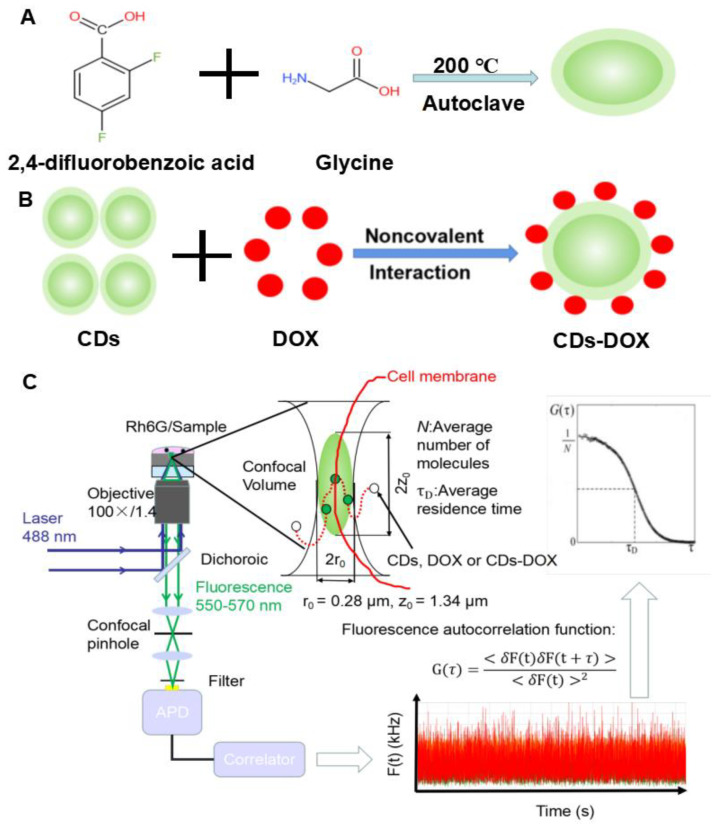
Schematic illustration of the preparation of CDs (**A**) and CDs-DOX (**B**) and the FCS methods to study for nanodrug transmembrane transport (**C**).

In this article, we report, for the first time, the study of CDs-DOX transmembrane delivery with FCS methods. A nanosized CDs material was first prepared by one-pot hydrothermal synthesis (Figure 1A) and incorporated with DOX to form the CDs-DOX composite (Figure 1B). Followingly, a kinetic model of mass transportation was set up and calibrated with FCS imaging method to describe the transmembrane delivery of drug in living cells. The transmembrane processes of bare DOX, CDs, and CDs-DOX composites were studied quantitatively to determine their different diffusion character with auto-correlation function (Figure 1C) and compared with the intensity information. On that basis, the plausible mechanism of transmembrane trafficking efficiency improvement was discussed. Finally, we used FCS to study the effects of ATP concentration and pH value on drug transport across the membrane, further showing the potential of applying this method for studying drug uptake in complex tumor microenvironments.

## 2. Materials and Methods

### 2.1. Materials and Characterization

Doxorubicin hydrochloride (DOX), RMPI 1640 and McCoy’s 5A were purchased from Shenzhen Ruixin Biological Technology Co., Ltd. (Hyclone, Shenzhen, China). HCl, 2,4-difluorobenzoic acid and glycine were obtained from Sigma-Aldrich Co., Ltd. (MACKLIN, Shenzhen, China). Human bone osteosarcoma epithelial cells (U_2_OS) and mammalian cells (COS7) were provided by Shenzhen Ruixin Biological Technology Co., Ltd. (Hyclone, Shenzhen, China). Dialysis bags (500 Da and 500 Da–1000 Da) were purchased from Shenzhen Maibeilai Technology Co., Ltd. (ACMEC, Shanghai, China). Ultra-pure water was produced in our laboratory. HCl was used to adjust the pH value of samples.

Transmission electron microscopy (TEM) images of the CDs, DOX and CDs-DOX were recorded by a tungsten filament transmission electron microscope (HITACH HT7700 A5-110, Tokyo, Japan). The particle sizes were measured with Nano Measurer software. A UV-visible spectrophotometer (Lambda 35, PerkinElmer, Waltham, MA, USA) was used to collect the UV-visible absorption spectra, and fluorescent spectra were obtained by a fluorescence spectrophotometer (F-4500, HITACH, Tokyo, Japan). Cell imaging was performed using a fluorescence lifetime imaging system (DCS-120, Becker & Hickl, GmbH, Berlin, Germany) with a supercontinuum laser (WhiteLase, Fianium, UK). In this study, all the images were obtained with an excitation wavelength of 488 nm, an oil immersion objective (100×, NA 1.40) and a bandpass filter (LP 495) to improve the signal-to-noise ratio.

### 2.2. CDs and CDs-DOX Preparation

One gram of 2,4-difluorobenzoic acid and 1.25 g of glycine were first dissolved in 25 mL ultrapure water. Then, the mixture was placed into a stainless-steel autoclave and carbonized at 200 °C for 48 h. After cooling to room temperature, the resultant solution was filtered with deionized water in a 500 Da dialysis bag to remove impurities [20,21].

DOX was bound to CDs through noncovalent interactions [22,23]. Briefly, 1 mL of DOX·HCl (0.4 mg/mL) was added to 1 mL of CDs (4 mg/mL) and then stirred for 24 h at 28 °C in the dark. Then the solution was dialyzed with a dialysis bag (MWCO = 500–1000 Da) for 6 h to obtain CDs-DOX. Finally, the CDs-DOX were stored at 4 °C for further processing.

### 2.3. Quantum Yield Measurement

Quantum yield (QY) measurements were performed according to the slope method [24,25]. Rohdmaine 6G dispersed in ethanol (QY 95%) was employed as a standard. The absorbance of the solution for green-emitting CDs and rhodamine 6G was kept below 0.06. The QY of the prepared green-emitting CDs was calculated according to the following Equation (1).

φ_x_ = φ_st_ × (K_x_/K_st_) (η^2^_x_/η^2^_st_), (1)

where φ is the quantum yield, K is the slope of the fitted line and η is the refractive index of the solvent. The subscript “x” refers to the testing sample and “st” refers to the standards (rhodamine 6G). The refractive index values are 1.33 and 1.36 for water and ethanol, respectively.

### 2.4. Cell Imaging

For CDs staining, 20 µL of CDs (4 µg/mL) was added to the cell culture plate (1 mL of the cell culture medium, the number of the U_2_OS and COS7 cells reached 60% confluence). The cell culture dish was shaken gently to spread the CDs evenly (less than 1 min), and the cells were immediately monitored by a fluorescence lifetime imaging system. DOX and CDs-DOX were stained under the same conditions as the CDs. Five minutes later, the drug was added to the cell culture plate; the images were collected at a rate of one frame per minute for the first 20 min, then at a rate of one frame per five minutes, thereafter. All image collection times were set to 15 s, and all experiments lasted for 1 h.

### 2.5. FCS Setup

U_2_OS and COS7 cells were seeded into cell dishes two days before the experiment, and grown to more than 50% confluency on cell culture dishes. Cells were exposed to 4 µg/mL CDs, DOX and CDs-DOX before starting the experiment. Then, FCS measurements were performed on a fluorescence lifetime imaging system based on an inverted confocal microscope (Eclipse, Nikon) with an objective lens (100×, NA 1.40) at room temperature and the laser power was 0.5 µW. We first studied the influence of the distance from the cover slip to the objective lens on the FCS data, such as the number of molecules in the micro-area, the diffusion time and the brightness of the molecules (Figure S1A and Figure 1B,C). It can be concluded that there is little change in the number of molecules in microdomain and the diffusion time when the distance is from −15 µm to 30 µm. When the distance is below −15 µm, the effective measurement volume will be mismatched. We ensured that the distance between the cover glass and the objective lens was within −15 µm to 30 µm to make accurate measurements. In this way, the FCS measurement volume was calibrated using a 200 nM rhodamine 6G (Figure S2) before each measurement, and the ratios r_0_ (0.28 µm) and z_0_ (1.34 µm) of the volume element were determined.

The data recording times were varied from 40 s to 60 s for both the rhodamine 6G solution and live cell samples. To perform the pH experiment, the HCl was added to adjust the pH value of the cell medium, and the pH value was measured with pH test paper and then samples were incubated for 10 min prior to the FCS experiment. In ATP experiments, cells were preincubated with ATP at different concentrations for 5 min prior to CDs, DOX and CDs-DOX addition. In all FCS measurement processes, the laser was always focused on the cell membrane to measure the drug transmembrane process.

The autocorrelation function G(τ)=〈δF(t)δF(τ+t)〉/〈F(t)〉^2^ was used to analyze the time-varying fluorescence intensity fluctuation caused by the diffusion of the fluorescent substance through the confocal observation volume. The fitting of the autocorrelation function of all experiments was performed with Burst Analyzer software (Becker & Hickl, GmbH, Berlin, Germany).

It was assumed that the focal volume is a three-dimensional Gaussian distribution, the analytical expression for the autocorrelation function of fluorescent species has the following form:

(2) $$G(\tau )=\frac{1}{N}(1+\frac{Te^{-\tau /\tau_{triplet}}}{1-T})(\frac{1}{1+\frac{\tau }{\tau_{D}}})\frac{1}{\sqrt{1+{\left(\frac{\omega_{0}}{z_{0}}\right)}^{2}\frac{\tau }{\tau_{D}}}},$$

where *N* is the average number of fluorescent particles in the excitation volume, and the concentration C can be obtained with the following equation:

(3) $$C=\frac{N}{V_{eff}}$$

*T* is the fraction of triplet fluorescent molecules with lifetime $\tau_{triplet}$, $\tau_{D}$ is the characteristic diffusion time of fluorescent molecules, and the characteristic diffusion time $\tau_{D}$ is defined as:

(4) $$\tau_{D}=\frac{\omega_{0}^{2}}{4D}$$

both the diffusion coefficient *D* and $\tau_{D}$ and the effective volume $V_{eff}$ (the spatial resolution of a confocal fluorescence microscope is usually described by the effective volume, that is the excitation volume folded with the detection volume) can be obtained by calibrating with a standard substance (such as rhodamine 6G).

The transmembrane diffusion was defined by the following equation:

(5) $$D_{transport}=\frac{\tau_{Rh6g}}{\tau_{transport}}*D_{Rh6g},$$

where *τ_Rh6g_* and *D_Rh6g_* are the diffusion time and diffusion coefficient in water, respectively. *τ_transport_* and *D_transport_* are the diffusion time and diffusion coefficient across the membrane, respectively.

### 2.6. Statistical Analysis

The results of all of the experiments are given as the mean values ± standard deviation (SD) obtained from multiple samples. FCS measurements were performed with triplicate samples in three independent experiments. For each sample, three to six measurements were taken. Data sets were considered significant when *p* < 0.05 with Student’s *t*-test [26].

## 3. Results

### 3.1. Synthesis and Characterization of CDs, DOX and CDs-DOX

**Figure 2:**
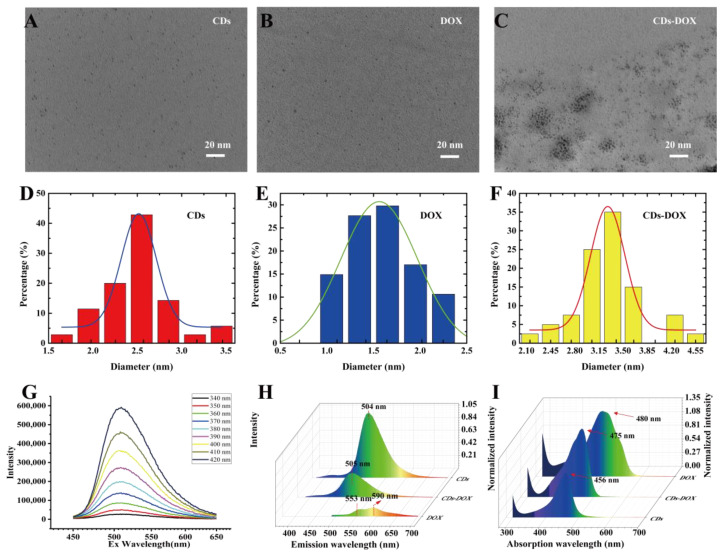
Morphology and characterization of CDs, DOX and CDs-DOX. (**A**–**C**) The TEM images of CDs, DOX and CDs-DOX. (**D**–**F**) The height distribution of CDs, DOX and CDs-DOX. The average heights of CDs, DOX, and CDs-DOX were approximately 2.5 nm, 1.5 nm and 3.5 nm, respectively. Additionally, one hundred particles were analyzed to prepare the size of each distribution histogram. (**G**) Emission wavelength (460–640 nm) spectra of CDs under different excitation wavelengths (340–420 nm). (**H**) The emission wavelengths of CDs and CDs-DOX were located at 504 nm and 505 nm, while DOX had two emission peaks: 553 nm and 590 nm. (**I**) The absorption peaks of CDs, DOX and CDs-DOX were located at 454 nm, 480 nm and 475 nm, respectively.

The CDs were successfully synthesized by a one-step hydrothermal method and the morphology was analyzed by TEM measurements (Figure 2A). We can see that the CDs particles are spherical and well dispersed with a diameter of approximately 2–3 nm (Figure 2D); the DOX particles are smaller than the CDs with an average size of approximately 1.5–2 nm (Figure 2B,E), and the CDs-DOX complexes have the largest size of the three particles with a diameter of approximately 3–3.5 nm (Figure 2C,F). The size difference between these particles provides evidence for the attachment of DOX to the CDs. The quantum yield (14.48%, Figure S3) of CDs was determined in deionized water under 420 nm excitation using rhodamine 6G (quantum yield 95%) solution as a reference. The optical properties of these particles are important for biological applications, especially biological imaging, and drug delivery. Therefore, we studied the fluorescence properties of the three particles via their fluorescence spectra. As shown in Figure 2G, as the excitation wavelength increases from 340 nm to 420 nm, the fluorescence intensity gradually increases at an emission wavelength of 500 nm, indicating excitation-independent emission, which can be useful in multicolor biological imaging applications [26]. As shown in Figure 2H, CDs and CDs-DOX had single emission peaks at 504 nm and 505 nm, but DOX exhibited two emission peaks at 553 nm and 590 nm. Additionally, the intensities of CDs and DOX are higher than that of CDs-DOX, indicating that DOX molecules may quench the fluorescence of CDs to a certain extent [27]. The decrease in peak intensity proves that DOX was successfully loaded onto the CDs. In addition, the excitation spectra of CDs, free DOX and CDs-DOX solutions were also collected (Figure 2I). The absorption spectrum of the DOX solution exhibited a peak at 480 nm, and those of CDs and CDs-DOX were located at 458 nm and 475 nm, respectively. The similar absorption wavelengths of DOX and CDs-DOX indicate that CDs were successfully attached to DOX.

### 3.2. Investigating Nanodrug Transmembrane Transport

**Figure 3:**
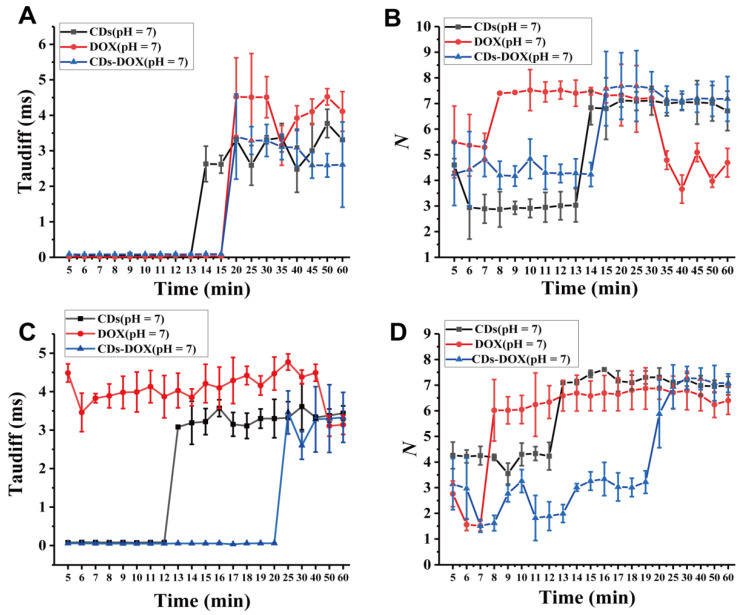
The diffusion times ($\tau_{D}$) and mean particle numbers (N) of the CDs, DOX and CDs-DOX in U_2_OS and COS7 cells at different times. (**A**,**B**) Diffusion times and mean particle numbers of the three particles in U_2_OS cells at different times. (**C**,**D**) Diffusion times and mean particle numbers of the three particles in COS7 cells at different times. Error bars are given as the mean values ± standard deviation (SD) obtained from multiple samples (n = 9).

To demonstrate that the FCS method can be used to monitor the transmembrane processes of CDs, DOX and CDs-DOX, we measured the diffusion times and the concentrations of the three particles in U_2_OS and COS7 cells at different time points. Figure 3A shows that CDs, DOX and CDs-DOX exhibit almost the same transmembrane diffusion curve under neutral conditions in U_2_OS cells. CDs, DOX and CDs-DOX exhibit a faster diffusion mode across the cell membrane during the first few minutes, which we call the free diffusion stage. CDs, DOX and CDs-DOX all exhibit hindered diffusion after 13, 15 and 19 min, respectively. For CDs, the free transmembrane diffusion mode occurred at 5 min with a diffusion coefficient of 9.32 ± 0.32 × 10^−^^6^ cm^2^ s^−1^ (*p* < 0.005) (Figure S4A,B). Compared with CDs at 5 min, DOX diffused faster (1.72 ± 0.1 × 10^−5^ cm^2^ s^−1^, *p* < 0.001, Figure S5A,B), and the free transmembrane stage lasted a longer time (Figure S6A). The main reason why the free transmembrane diffusion coefficient of DOX is larger than that of CDs is the smaller particle size of DOX. It is not surprising that CDs-DOX has the smallest diffusion coefficient at 5 min (3.89 ± 0.14 × 10^−6^ cm^2^ s^−1^, *p* < 0.001, Figure S7A,B) as it has largest particle size. To quantify the performance of the nanodrugs in cells, we measured the mean particle number *N* (Figure 2B), in which we obtained the concentrations of the nanodrugs in cells according to Equation (3). As shown in Figure 3B, the concentrations of CDs and CDs-DOX were low during the first 14 min, then increased and remained stable, while that of DOX increased, then remained stable, and finally decreased. It is worth to mentioning that the concentration of CDs-DOX (188.35 ± 13.42 nM) is higher than that of CDs and DOX after 30 min (Figure 3B), indicating that the drug delivery system of CDs-DOX is more stable and effective than DOX alone. The different concentrations of CDs and DOX can be ascribed to different cellular uptake mechanisms: cells absorb DOX through passive diffusion, while CDs enter cells through endocytosis and passive diffusion [28]. The combination of the two pathways may explain the high concentration and longer free diffusion time of CDs-DOX in cells [29].

To verify whether the drug delivery system has similar transmembrane transport characteristics with regard to other cells, we performed the same experiment with COS7 cells (Figure 3C,D). As shown in Figure 3C, CDs and CDs-DOX exhibited transmembrane curves similar to those with U_2_OS cells. The free diffusion processes for CDs and CDs-DOX lasted 12 min and 20 min, respectively, (Figure S6B). Moreover, the diffusion coefficient of CDs-DOX (5.94 ± 0.9 × 10^−6^ cm^2^ s^−1^, *p* < 0.005, Figure S8A,B) was higher than that of CDs (4.03 ± 0.3 × 10^−6^ cm^2^ s^−1^, *p* < 0.001, Figure S9A,B). Surprisingly, DOX did not show a free diffusion process in COS7 cells, and the transmembrane process was hindered at the beginning (Figure S10A,B). This may because U_2_OS are cancer cells that express more carrier proteins on the cell membrane surface than COS7 cells [30]. In addition, the highest concentrations (201.94 ± 11.65 nM at 30 min, *p* < 0.05) of CDs-DOX in COS7 cells after 30 min were also observed compared with CDs and DOX (Figure 3D).

To further interpret the FCS data, we performed confocal imaging on U_2_OS and COS7 cells. Figure S11 shows the fluorescence imaging of the CDs, DOX and CDs-DOX in the U_2_OS cells. The free CDs were mainly distributed in the cytoplasm between 5 min and 12 min, and most of the CDs are gathered in the nucleus. DOX accumulates on the cell membrane surface within 5–14 min, and then DOX gathers in the nucleus. Surprisingly, CDs-DOX was observed in the cytoplasm and nucleus for the first time, which may be ascribed to the fact that DOX-CDs enter cells through endocytosis and passive diffusion, which is also consistent with the FCS results that indicate CDs-DOX has a longer free diffusion stage, and ultimately achieves a higher concentration in U_2_OS cells. In COS7 cells (Figure S12), DOX gathered in the cell membrane for the first few minutes (5–15 min), and was then observed in the cytoplasm and nucleus. However, CDs and CDs-DOX accumulate in the nucleus at 5 min, demonstrating that CDs can promote the rapid entry of DOX into the cell membrane.

The low-speed diffusion of CDs (Figures S5C and S10C) and the increase in the number of molecules microdomains may be caused by the following reasons: First, CDs enter cells through free diffusion and passive diffusion. The diffusion speed is related not only to the concentration of CDs, but also to the number of carrier proteins on the cell membrane. In the low diffusion period of CDs, the decrease in CDs concentration leads to a decrease in diffusion speed; additionally, CDs are bound by a large number of carrier proteins, which increases the number of CDs molecules detected in the micro-domain [31]. Second, CDs enter the cell through endocytosis, and an endocytic cycle may occur in the cell: part of the CDs enters the lysosome for degradation; part is sent to the plasma membrane through the recycling mechanism, and; part is finally sent to the nucleus. In the slow diffusion fraction, the recovery mechanism may be dominant, and most of the CDs are delivered to the plasma membrane, which increases the number of CDs in the microdomain [32]. Third, in some cases, nanoparticles may create nanosized pores in the membrane to pass through it. The endocytosed nanoparticles are confined in the endosomes and may not be able to leave the endosomes and reach the cytoplasm. This may cause the accumulation of CDs on the cell membrane and limit the spread of CDs across the membrane [33].

The transport of nanoparticles across the membrane is related to the interaction between the charged particles and the cell membrane, hydrophobicity of the nanoparticles, and the endocytosis of the cell membrane. In our experiments, the nucleus was stained with CDs after 30 min. In addition, the nucleus mainly contains DNA and RNA, indicating that CDs have an affinity for DNA and RNA. Since DNA and RNA are negatively charged, it can be assumed that CDs are positively charged [34]. For the same reason, the CDs-DOX are also positively charged. The cell membrane is negatively charged, so it is not surprising that CDs and CDs-DOX diffuse faster than DOX. DOX binds to nucleic acids, presumably by specific intercalation of the planar anthracycline nucleus with the DNA double helix [35]. The anthracycline ring is amphoteric, which indicates that DOX can quickly bind to and pass through the cell membrane. Meanwhile, nanomaterials can pass through cell membranes through endocytosis, and the shape of nanoparticles greatly affects their cellular uptake [36]. As shown in Figure 1A–C, our material is similar to spherical particles and thus has a fast diffusion coefficient.

FCS and cell-imaging data quantitatively and quantitatively illustrate that CDs-DOX is an effective drug delivery system in cells, and the high concentration of DOX drugs delivered into cancer cells may improve the therapeutic ability of DOX toward tumor cells.

### 3.3. Investigating Nanodrug Transmembrane Transport under Different Conditions

The pH value plays a role in controlling DNA synthesis, cell proliferation, the protein synthesis rate, cellular proliferation, and cell volume regulation [37]. In addition, ATP is an energy currency that is ubiquitous in all living organisms, and the high phosphate transfer potential of ATP can be used in many biological processes, including muscle contraction, biomolecule synthesis and membrane transport [38]. Therefore, it is necessary to study the influence of pH value and ATP concentration on drug transport across the membrane. The pH value of a tumor cell (pH 6.5–7.2) is slightly lower than that of normal tissues and blood (pH 7.4). Meanwhile, the pH values in endosomes (pH 5.5–6.0) and lysosomes (pH 4.5–5.0) are even lower [39], so we chose different pH values (pH = 5, 6, 7) to study their influence on the process of drug transmembrane transport. Because a higher ATP concentration (over 100 µM) will affect cell membrane permeability [40], two APT concentrations of 50 µM and 500 µM were selected to study the impact on transmembrane transport in U_2_OS cells.

**Figure 4:**
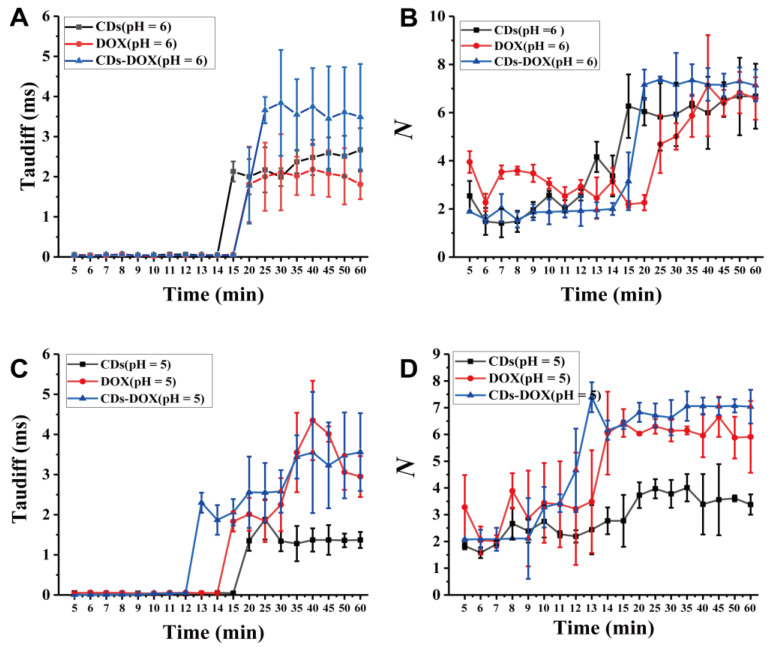
The diffusion time and the number of particles of CDs, DOX and CDs-DOX diffusing across the U_2_OS cell membrane at pH 6 (**A**,**B**) and pH 5 (**C**,**D**). HCl was added to adjust the pH value of the cell medium, and the pH value was measured with pH test paper, followed by incubation for 10 min prior to FCS experiments. Error bars represent the standard deviation of 9 measurements.

Figure 4A shows the diffusion times of CDs, DOX and CDs-DOX at pH = 6. Under this condition, it seems that the free diffusion times and the diffusion coefficients at this stage of CDs, DOX and CDs-DOX are almost the same (Figure S13A). Meanwhile, DOX has the highest diffusion coefficient, and CDs-DOX diffuses the most slowly after 20 min, which may provide a clue regarding the higher cellular uptake of CDs-DOX (Figure 4B). It is worth mentioning that the diffusion coefficient of CDs-DOX is almost 5 times larger than that of CDs and DOX from 5 to 8 min, indicating that CDs-DOX diffuses faster under acidic conditions (Figures S13B and S14, and Table 1). In addition to these factors influencing the transmembrane processes of CDs, DOX and CDs-DOX, it is worth mentioning that the data in Table 1 was measured under acidic conditions (pH = 5). It has been reported that lowering the extracellular pH of cells stimulates the formation of intima and vesicles while increasing the absorption of macromolecules [41], which can further explain the quick diffusion of CDs, DOX and CDs-DOX. In addition, the obstructed transmembrane transport of DOX gradually increases from 14 to 40 min and then begins to weaken, while the hindered diffusion transport of CDs remains almost stable and that of CDs-DOX increases first and then is almost stable. Even under acidic conditions, CDs-DOX showed the highest concentration (195.839 ± 8.88 nM, *p* < 0.001) in U_2_OS cells after 30 min (Figure 4D).

To understand the relationship between the transmembrane transport of CDs, DOX and CDs-DOX and pH, we drew the diffusion time diagrams of CDs, DOX and CDs-DOX at different pH values (Figure S15). The diffusion coefficient of CDs generally increases with increasing pH value, but the length of the free diffusion phase is reversed (Figure S15A). However, the diffusion coefficient at pH = 7 is twice times that at pH = 5 and pH = 6, which may explain why the free diffusion time is shortened. The free diffusion time of DOX has no significant response to pH values, and faster diffusion of DOX occurred at neutral condition (Figure S15B), because uncharged DOX penetrates through cell membranes faster than its protonated cationic form (DOX^+^) [40]. Meanwhile, the length of the free diffusion stage of CDs-DOX decreased as the pH increased, and CDs-DOX tended to diffuse faster under acidic conditions (Figure S15C).

**Figure 5:**
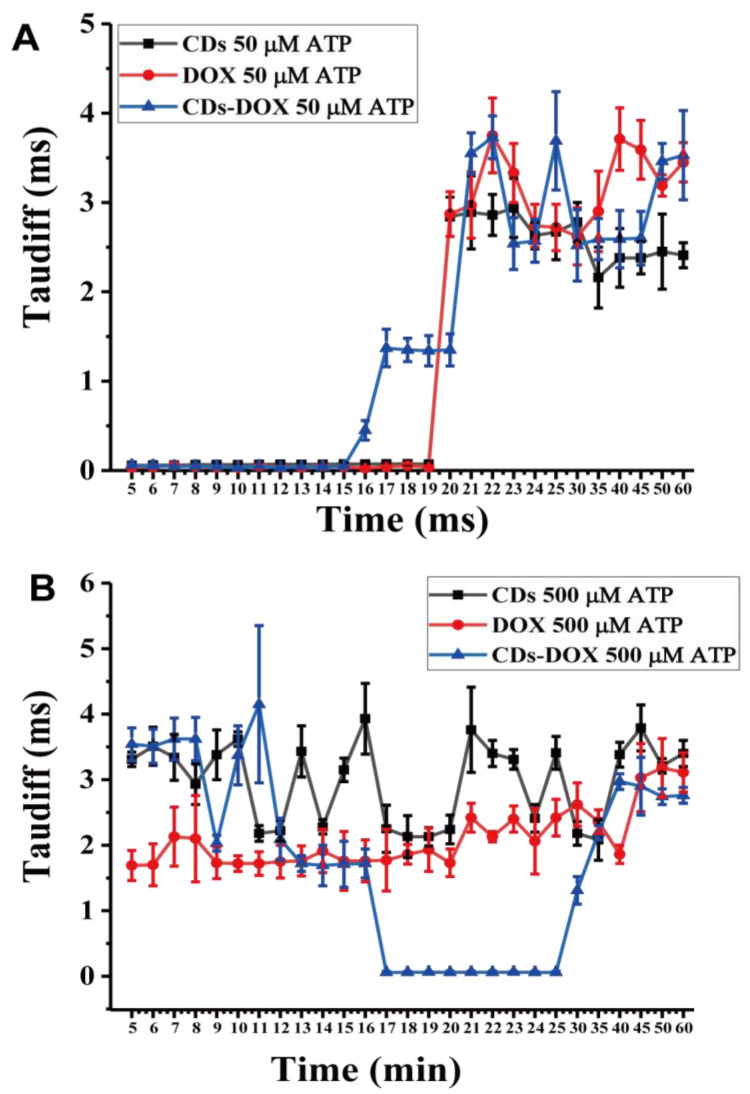
The effect of ATP concentration on drug transmembrane transport. The diffusion times changed with time when the ATP concentrations were 50 µM (**A**) and 500 µM (**B**). Cells were preincubated with 50 μL ATP at different concentrations and for 5 min prior to CDs, DOX and CDs-DOX addition. Error bars are given as the mean values ± standard deviation (SD) obtained from multiple samples (n = 9).

Furthermore, the ATP concentrations were also taken into consideration. As shown in Figure 5A, the CDs, DOX and CDs-DOX transmembrane processes were measured at an ATP concentration of 50 µM. As predicted, the transmembrane curves of CDs, DOX and CDs-DOX were similar to those without ATP (Figure 3A). CDs, DOX and CDs-DOX crossed the membrane slowly during the first 16 min when the ATP concentration was 500 µM (Figure 5B). Moreover, it is interesting that CDs and DOX initially exhibit hindered transmembrane diffusion, while the CDs-DOX free diffusion process (5.45 ± 0.908 × 10^−6^ cm^2^ s^−1^, *p* < 0.005, n = 9) is delayed. When the ATP concentration is over 100 µM, it binds to the P_2_X_7_ receptor on the cell membrane surface to increase membrane permeability [40], which may explain why the three particles suffer from hindered diffusion. Additionally, DOX accumulates specifically in mitochondria at a concentration 100 times higher than that observed in other tissues [42]. As CDs-DOX has two diffusion pathways across the membrane, CDs-DOX may rapidly accumulate in mitochondria, causing mitochondrial fission. Since mitochondria have been destroyed, cells absorb ATP added to the cell dish, and the ATP concentration may decrease to less than 100 µM; thus, CDs-DOX starts to diffuse rapidly.

## 4. Conclusions

In both U_2_OS and COS7 cells, the qualitative confocal imaging results were essentially consistent with the quantitative FCS results, demonstrating the feasibility of the FCS method for measuring CDs-DOX drug delivery systems across membranes. Additionally, compared with free DOX under acidic conditions, the CDs-DOX complex has higher cellular uptake and better transmembrane efficacy for U_2_OS cells. Under acidic conditions, DOX combines with H^+^ to form DOX^+^, resulting in low cellular drug uptake, which can provide different drug dosages for cells at different pH values. At low concentrations (~50 µM) of ATP, the diffusion coefficients of these three particles did not change significantly. However, high concentrations of ATP caused changes in cell membrane permeability, and hindered the transmembrane transport of CDs and DOX, thus delaying the rapid diffusion stage of CDs-DOX. The results of this study show that the FCS method can be used to study the expansion and transport of drugs in living cells and may provide a drug delivery strategy for the treatment of tumors in vivo.

## Figures and Tables

**Table 1:** Summary of the mean diffusion coefficients of CDs, DOX and CDs-DOX with standard errors on the mean at pH 5 from 5 min to 8 min (n = 9). “*” indicates that at the level of *p* < 0.05, there is no significant difference between the overall mean and the test mean. “**” indicates that at the level of *p* < 0.001, there is no significant difference between the overall mean and the test mean. “***” indicates that at the level of *p* < 0.001, there is no significant difference between the overall mean and the test mean.

Time (min)		5 min	6 min	7 min	8 min
Drug Diffusion Coefficient (×10^−6^ cm^2^ s^−1^)	CDs	5.91 ± 1.29 (*)	6.412 ± 1.25 (*)	7.32 ± 0.81 (**)	6.95 ± 1.89 (*)
	DOX	7.90 ± 1.15 (*)	5.5 ± 0.563 (***)	5.94 ± 0.32 (***)	5.94 ± 1.98 (*)
	CDs-DOX	69.58 ± 9.48 (**)	65.41 ± 10.9 (**)	46.72 ± 10.5 (*)	32.7 ± 10.9 (*)

## Data Availability

The data presented in this study are available on request from the corresponding author.

## Supplementary Materials

The following are available online at https://www.mdpi.com/article/10.3390/membranes11110891/s1, Figure S1: The influence of the distance between the cover glass and the objective lens on the detection volume of the number of molecules, Figure S2: Rhodamine 6G (SNR = 5 kHZ) diffusion time in aqueous solution, Figure S3: Plots of integrated intensity of CDs and rhodamine 6G as a function of optical absorbance at 420 nm and relevant data in deionied water, Figure S4: The information of the FCS measurements of CDs in U2OS Cells, Figure S5: Transmembrane process of FCS measurements of DOX, Figure S6: Free diffusion stage of CDs, DOX and CDs-DOX in U2OS, Figure S7: Transmembrane process of FCS measurements of CDs-DOX, Figure S8: Transmembrane process of FCS measurements of CDs-DOX, Figure S9: Transmembrane process of FCS measurements of CDs, Figure S10: Transmembrane process of FCS measurements of DOX, Figure S11: Confocal images of CDs, DOX and CDs-DOX in U2OS cells from 5 to 60 min, Figure S12: Confocal images of CDs, DOX and CDs-DOX in COS7 cells from 5 to 60 min, Figure S13: Transmembrane transport of CDs, DOX and CDs-DOX, Figure S14: The diffusion time of CDs, DOX, CDs-DOX, Figure S15: Transmembrane transport of CDs, DOX and CDs-DOX under different pH values.
