# Supplementary material for: Nanodrug Transmembrane Transport Research Based on Fluorescence Correlation Spectroscopy
Source: Membranes (Basel). 2021 Nov 19;11(11):891. doi: 10.3390/membranes11110891 (PMC8625013; doi:10.3390/membranes11110891)

membranes

Supplementary Information

Nanodrug Transmembrane Transport Research Based on Fluorescence Correlation Spectroscopy

Xinwei Gao, Yanfeng Liu, Jia Zhang, Luwei Wang, Yong Guo, Yinru Zhu, Zhigang Yang, Wei Yan * and Junle Qu

Center for Biomedical Photonics, Key Laboratory of Optoelectronic Devices and Systems of Ministry of Education and Guangdong Province, College of Physics and Optoelectronic Engineering, Shenzhen University, Shenzhen 518060, China; 1910454012@email.szu.edu.cn (X.G.); szu_liuyanfeng@163.com (Y.L.); julyzhang2021@163.com (J.Z.); wanglowell@szu.edu.cn (L.W.); 1800284004@email.szu.edu.cn (Y.G.); 2150453014@email.szu.edu.cn (Y.Z.); zhgyang@szu.edu.cn (Z.Y.); jlqu@szu.edu.cn (J.Q.)

* Correspondence: weiyan@szu.edu.cn

Figure S1.The influence of the distance between the cover glass and the objective lens on the detection volume of the number of molecules(A), the diffusion time(B), and the brightness(C). The zero points is the position where the clear image of the cell is found in the image mode. The distance between the objective lens and the cover glass is defined as positive when the objective lens moves forward relative to the zero points, otherwise, it is negative. Error bars represent the standard deviation of 5 measurements.

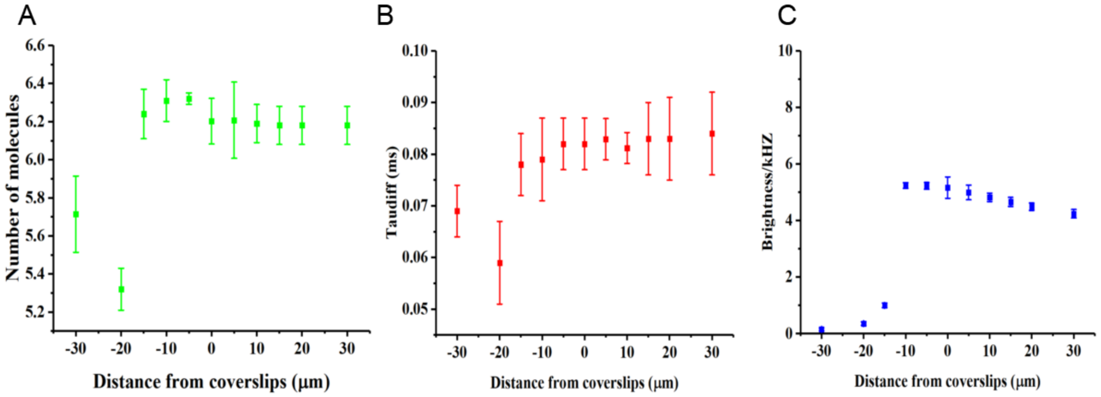

Figure S2. Rhodamine 6G (SNR = 5kHZ) diffusion time in aqueous solution.

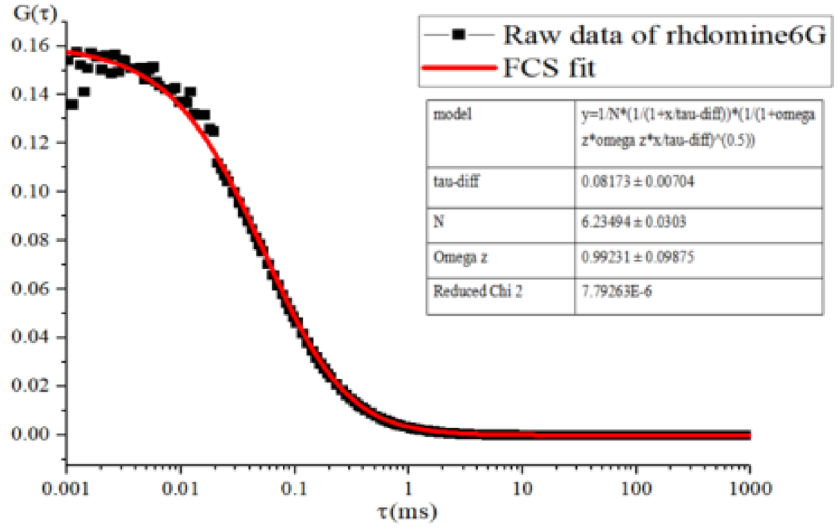

Figure S3. Plots of integrated intensity of CDs and rhodamine 6G as a function of optical absorbance at 420 nm and relevant data in deionied water.

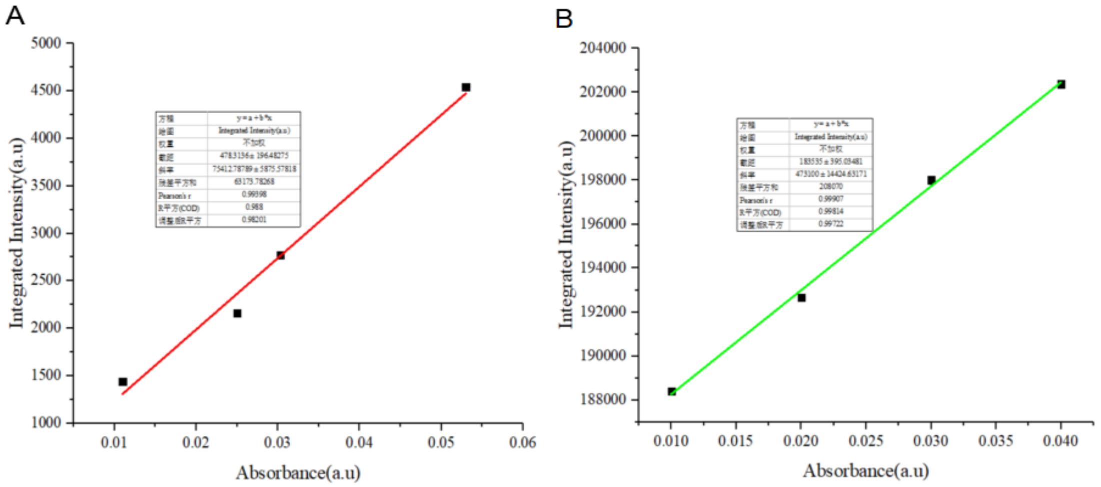

	CDs				Rhodamine 6G			
Abs	0.011	0.0025	0.0303	0.053	0.011	0.021	0.0303	0.0405
Integrated Intensity	1440	2160	2770	4540	188390	192670	198810	202380
Excitation (λ_{ex})	420 nm				420 nm			
Slope	75412				473100			
QY (%)	14.48				95			

Figure S4. The information of the FCS measurements of CDs in U2OS Cells. (A) The measurement position (yellow mark) in U2OS cells of CDs. (B, C) The raw data of FCS measurements in U2OS at 5 min (B) and 30 min(C), respectively. The scale bar is 10 μm.

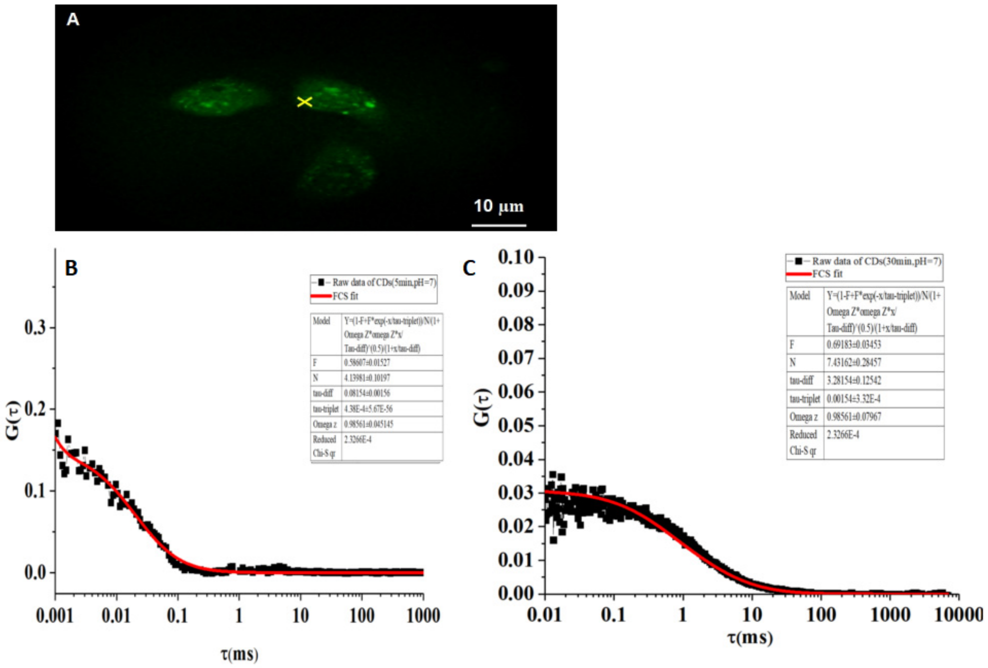

Figure S5. Transmembrane process of FCS measurements of DOX .(A) The measurement point of FCS in U2OS Cells, the green mark present measurement position.(B, C) The raw data of FCS measurement at 5 min(B) and 30 min(C), respectively.

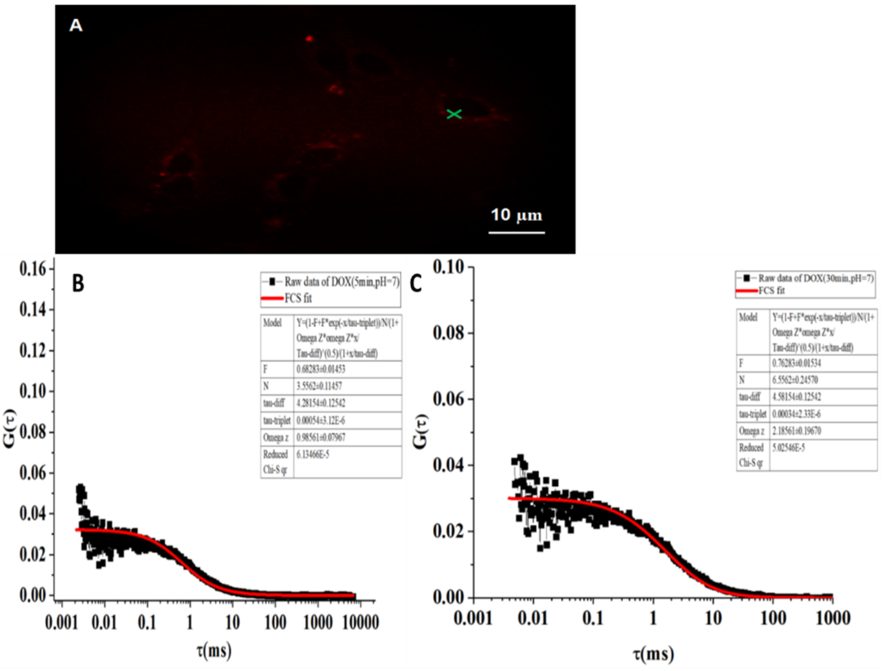

Figure S6. Free diffusion stage of CDs, DOX and CDs-DOX in U2OS (A) and COS7 (B) Cells.

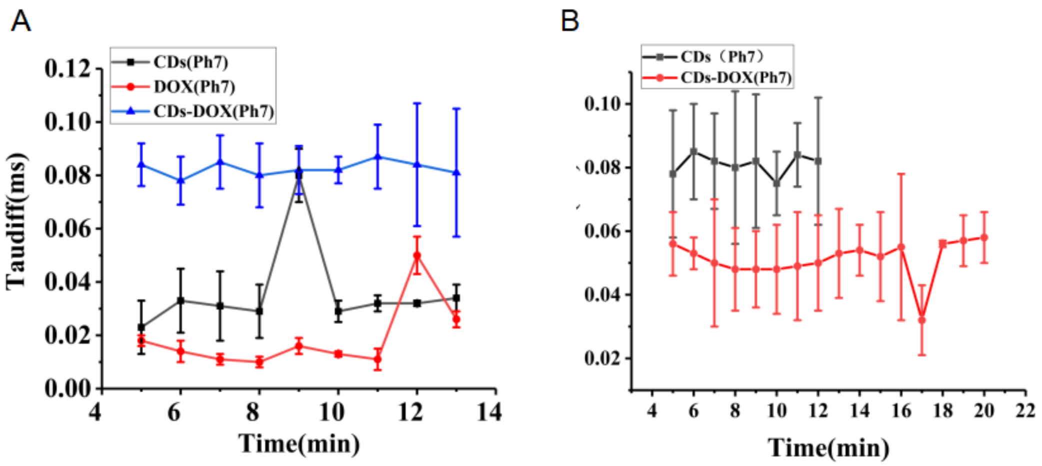

Figure S7. Transmembrane process of FCS measurements of CDs-DOX .(A) The measurement point of FCS in U2OS Cells, the red mark present measurement position.(B, C) the raw data of FCS measurement at 5 min(B) and 30 min(C).

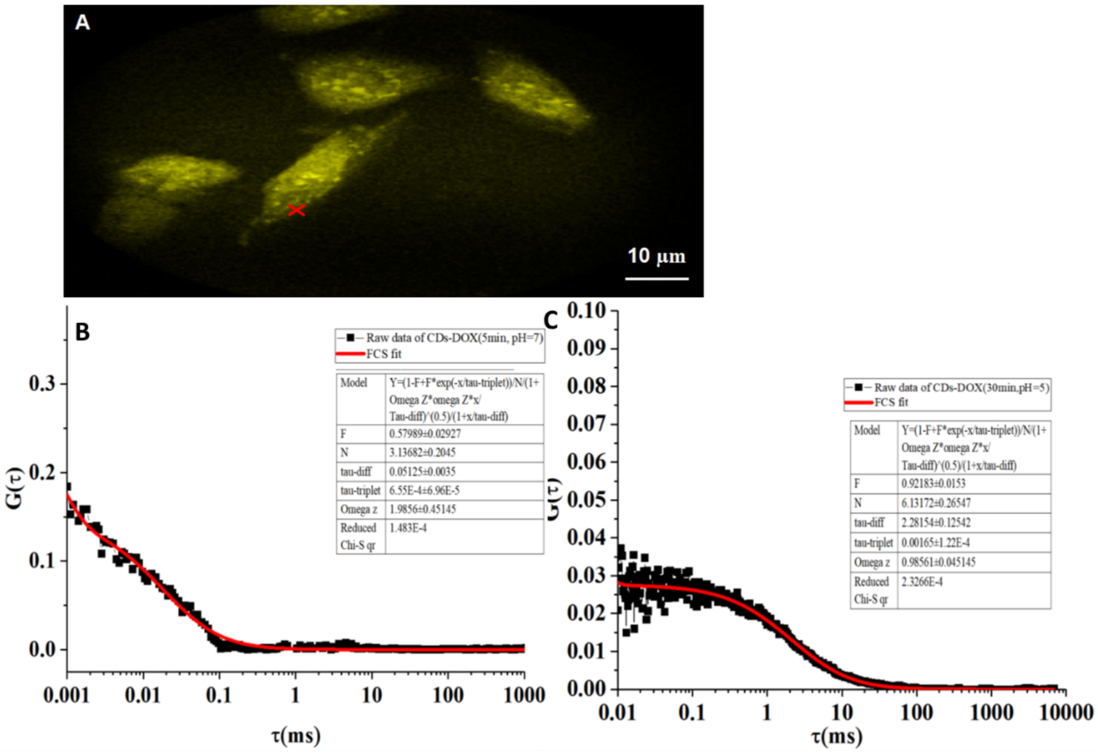

Figure S8. Transmembrane process of FCS measurements of CDs-DOX.(A) the measurement point of FCS in COS7 Cells, the red mark presents measurement position.(B, C) the raw data of FCS measurement at 5 min(B) and 30 min(C).

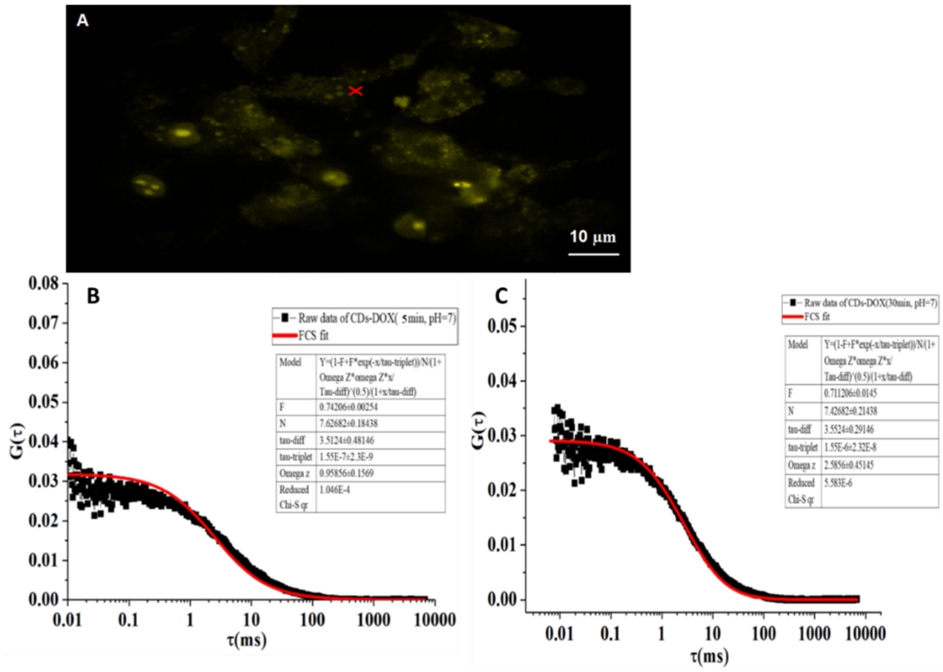

Figure S9. Transmembrane process of FCS measurements of CDs.(A) The measurement point of FCS in COS7 Cells, the green mark presents measurement position.(B, C) Data of FCS measurement at 5 min(B) and 30 min(C), respectively.

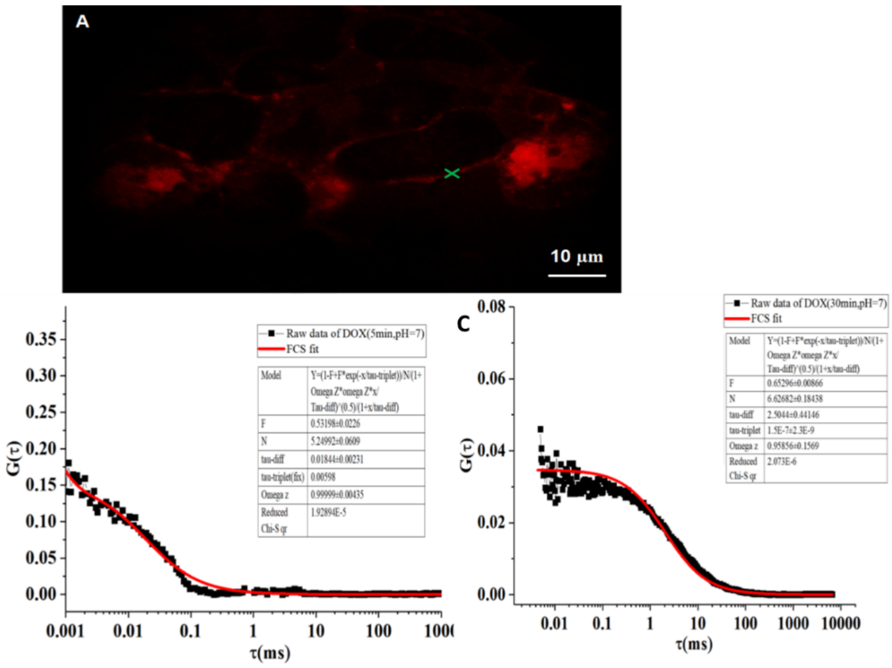

Figure S10. Transmembrane process of FCS measurements of DOX.(A) The measurement point of FCS in COS7 Cells, the green mark presents measurement position.(B, C) Data of FCS measurement at 5 min(B) and 30 min(C), respectively.

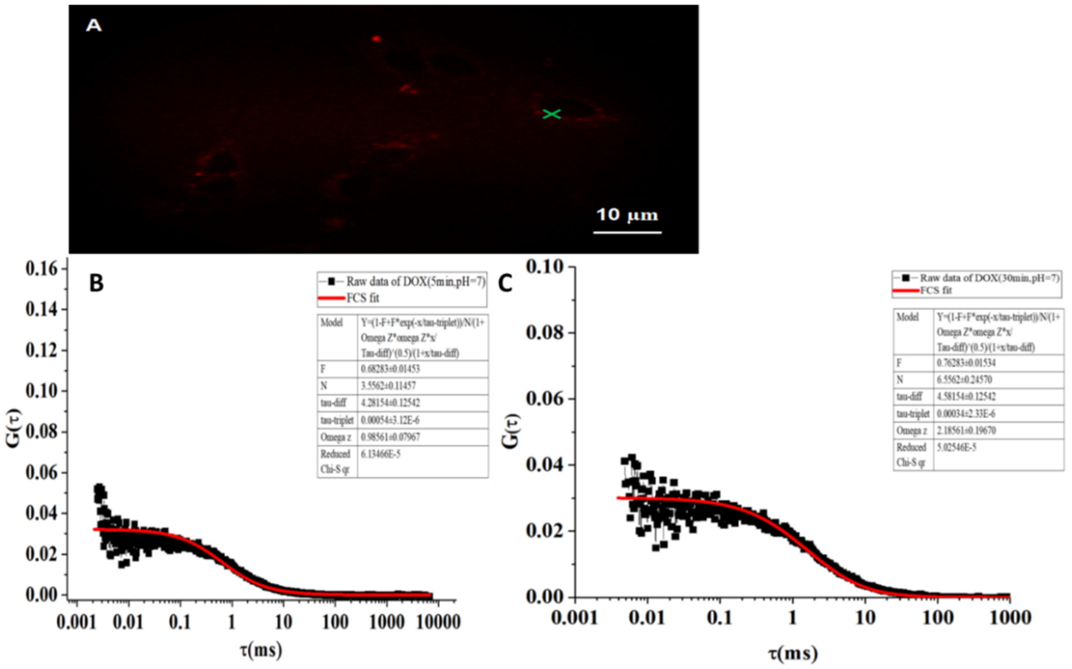

Figure S11. Confocal images of CDs, DOX and CDs-DOX in U2OS cells from 5 to 60 minutes. Scale bar is 10 μm.

CDs:

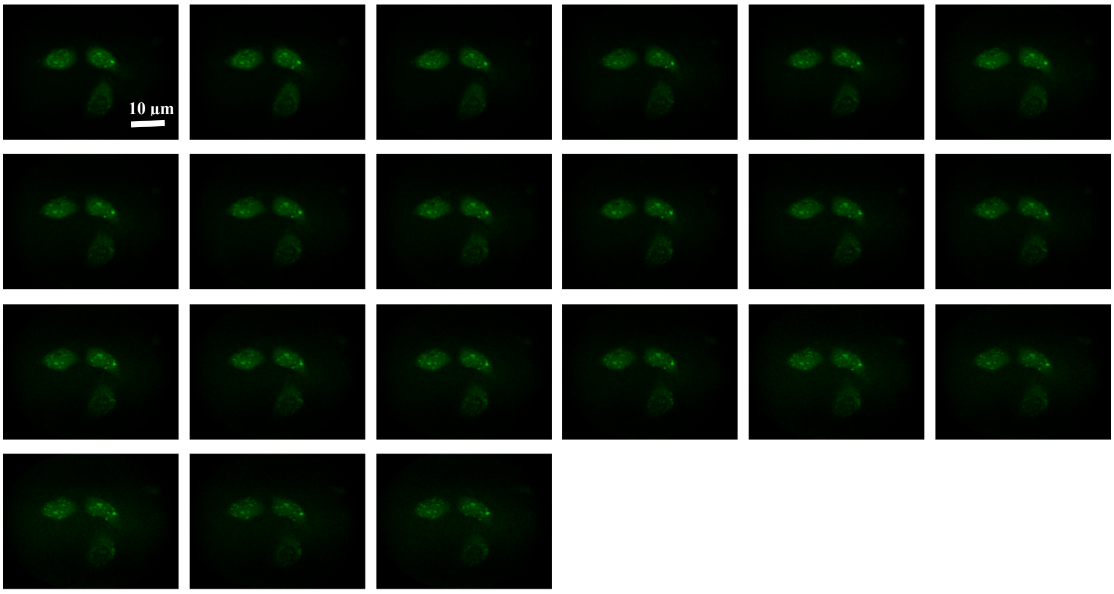

DOX:

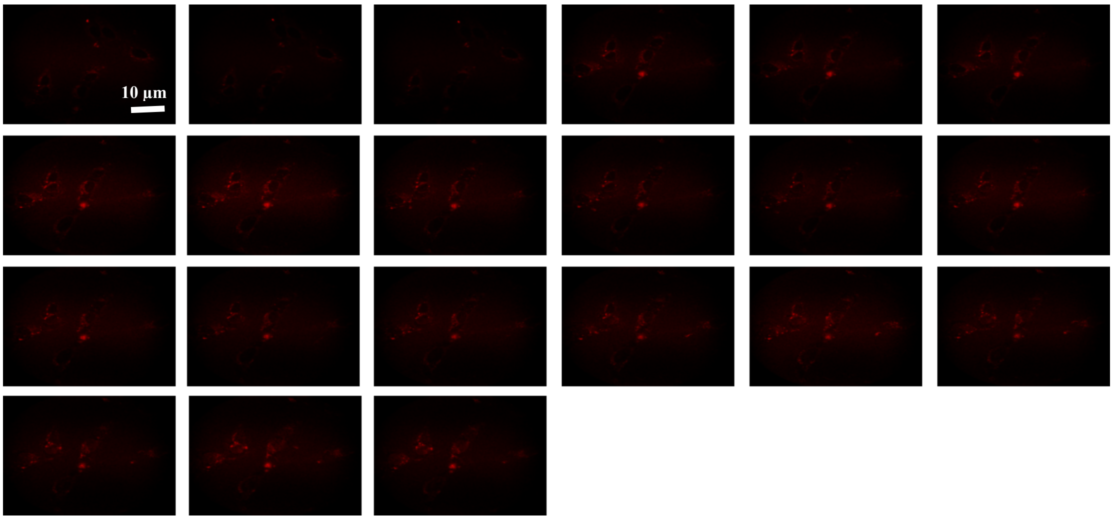

CDs-DOX:

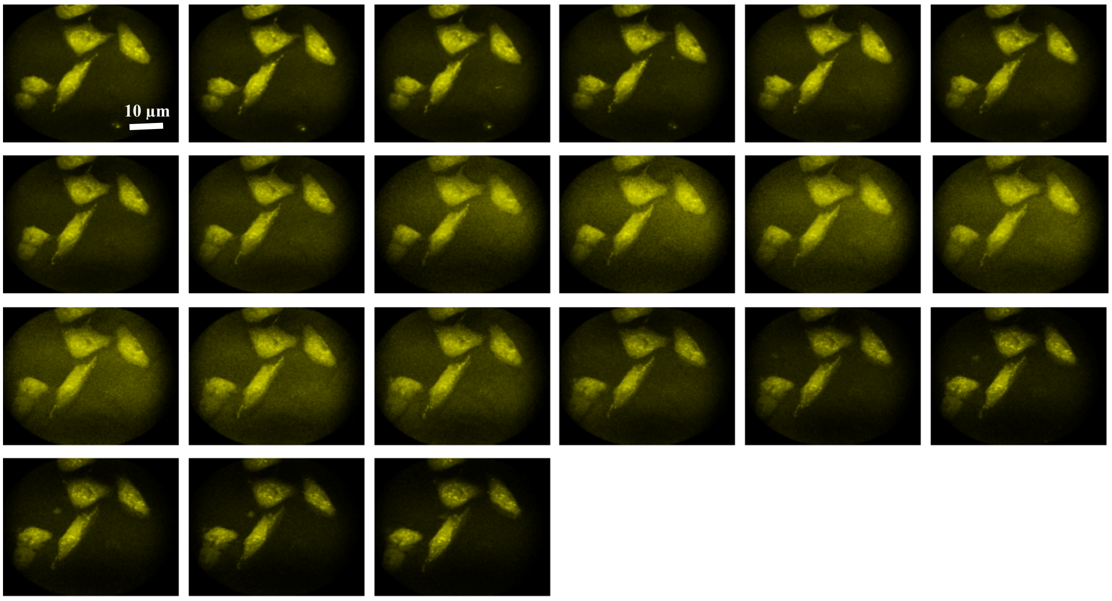

Figure S12. Confocal images of CDs, DOX and CDs-DOX in COS7 cells from 5 to 60 minutes. Scale bar is 10 µm.

CDs:

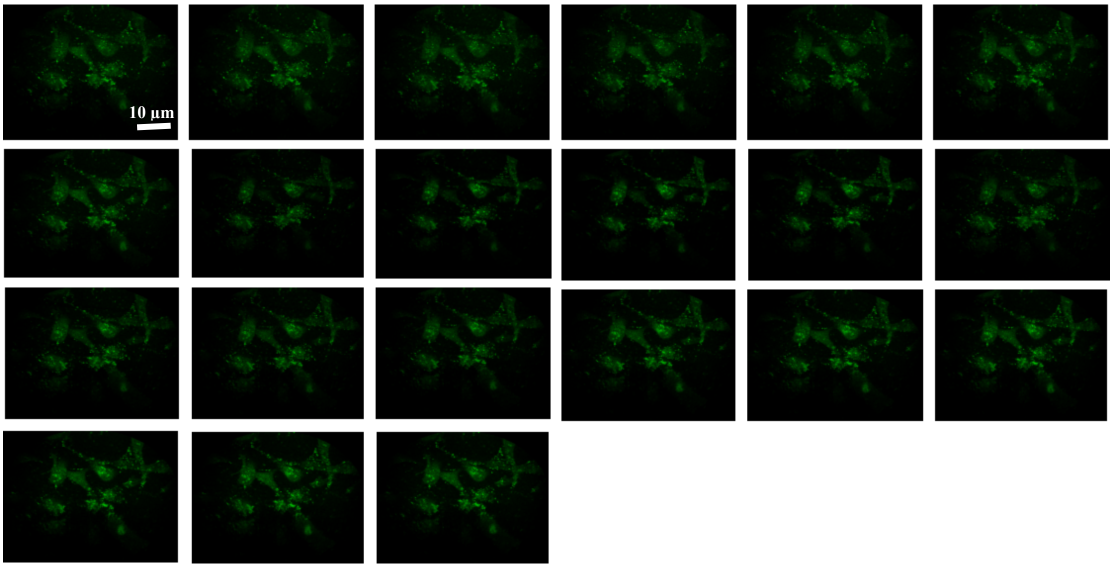

DOX:

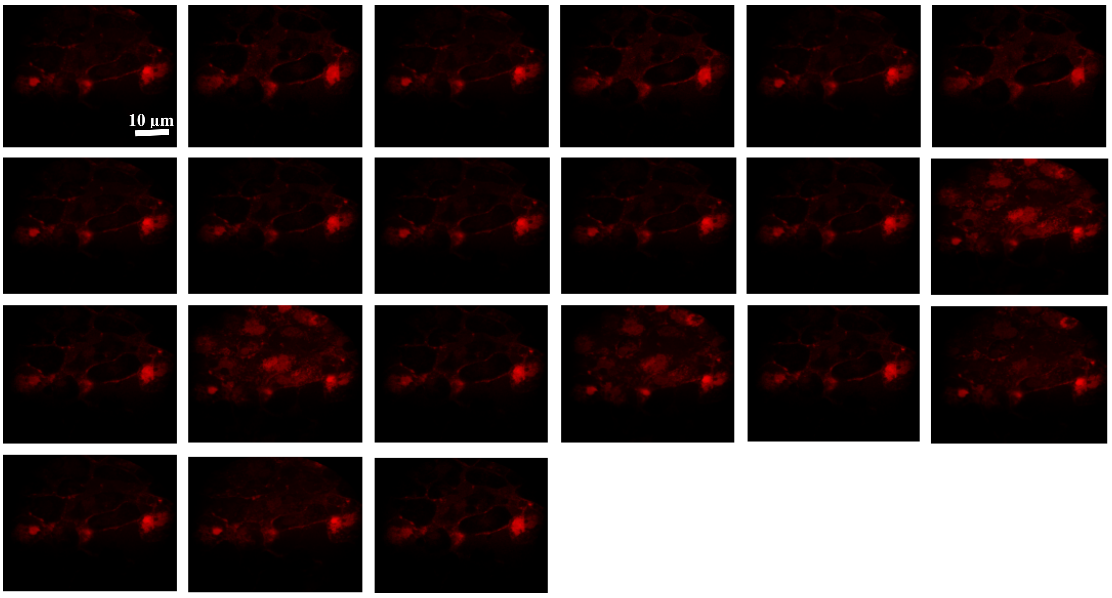

CDs-DOX:

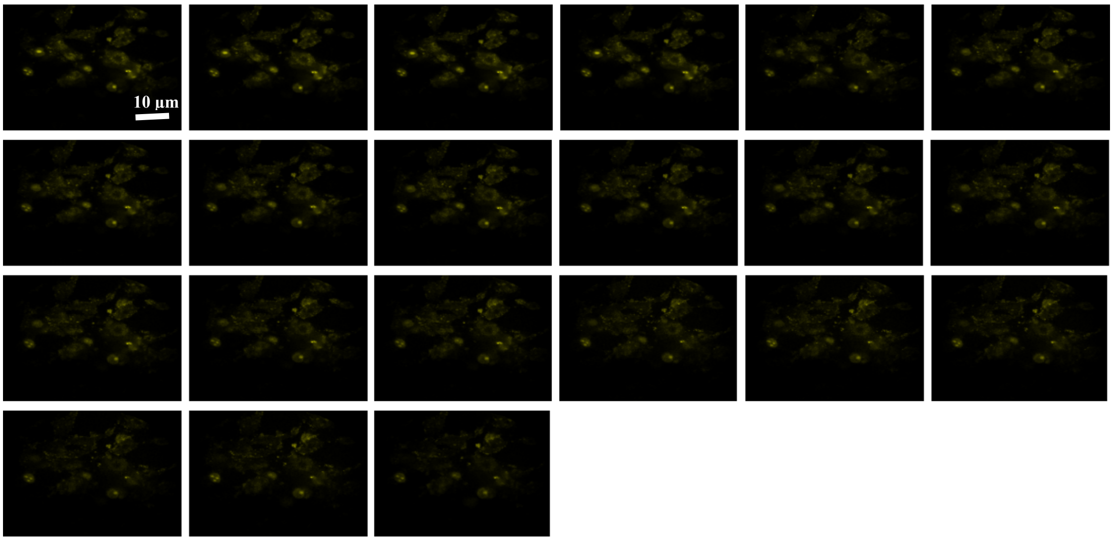

Figure S13. Transmembrane transport of CDs, DOX and CDs-DOX at pH6 and pH5 from 5 to 16 minutes.

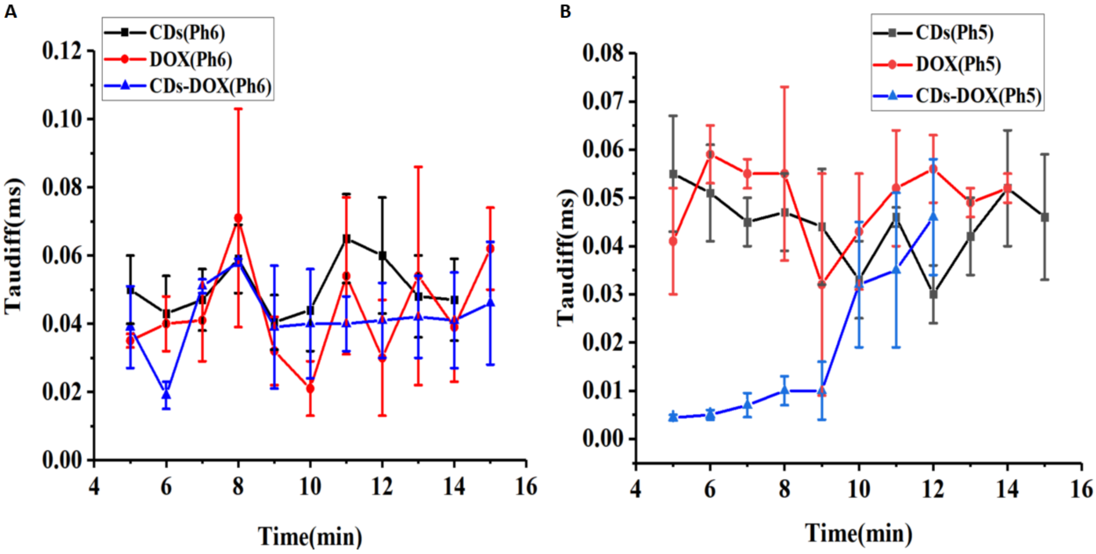

Figure S14. The diffusion time of CDs, DOX, CDs-DOX at pH = 5 from 5 min to 8 min in U_2Os cells.

CDs:

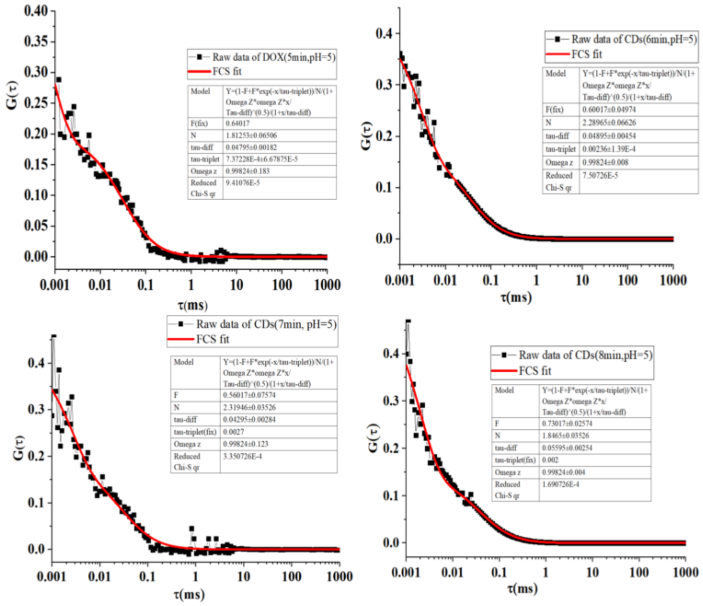

DOX:

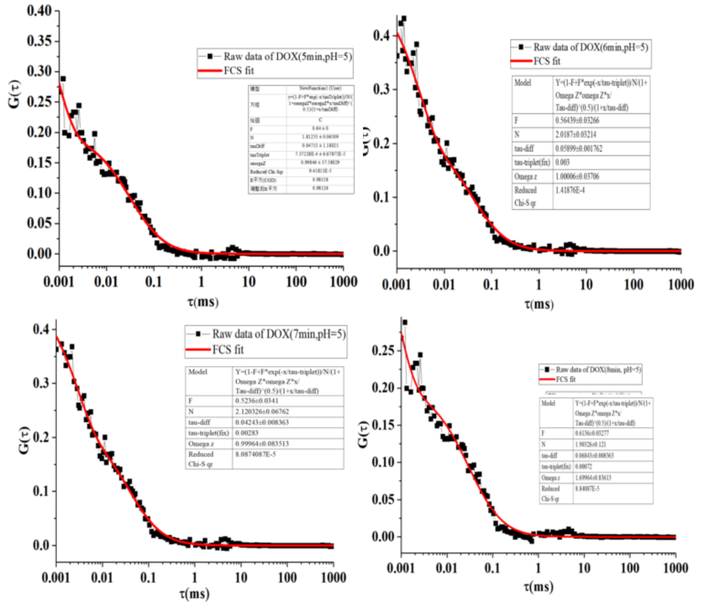

CDs-DOX:

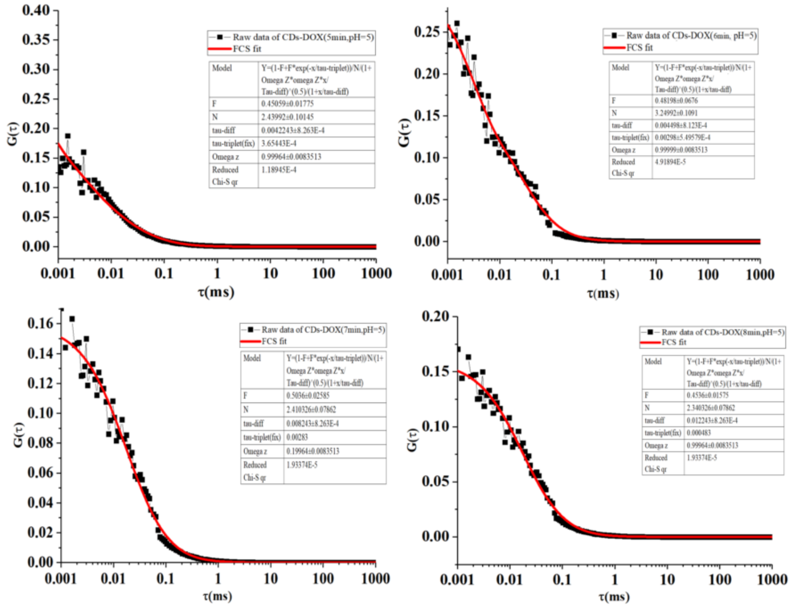

Figure S15. Transmembrane transport of CDs, DOX and CDs-DOX under different pH values.

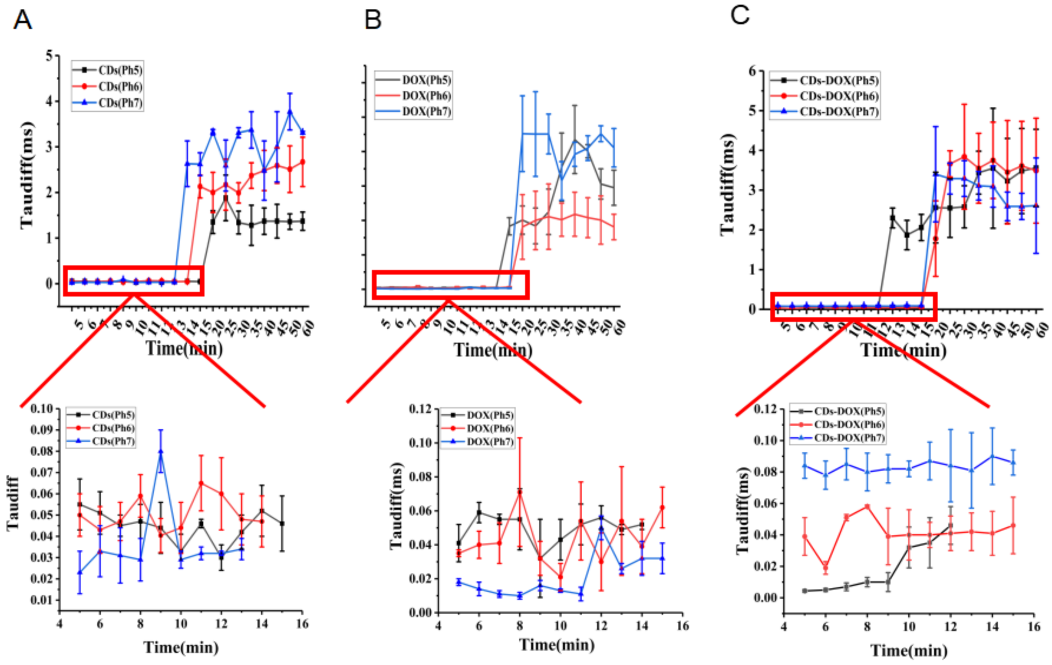

Supplement: Supplementary file 1 [file membranes-11-00891-s001.zip › membranes-1439162-SI.pdf]
